# Assessment of Mechanical/Chemical Properties and Cytotoxicity of Resin-Modified Glass Ionomer Cements Containing Sr/F-Bioactive Glass Nanoparticles and Methacrylate Functionalized Polyacids

**DOI:** 10.3390/ijms241210231

**Published:** 2023-06-16

**Authors:** Wisitsin Potiprapanpong, Parichart Naruphontjirakul, Chutikarn Khamsuk, Somruethai Channasanon, Arnit Toneluck, Siriporn Tanodekaew, Naruporn Monmaturapoj, Anne M. Young, Piyaphong Panpisut

**Affiliations:** 1 Faculty of Dentistry, Thammasat University, Pathum Thani 12120, Thailand; 2 Biological Engineering Program, Faculty of Engineering, King Mongkut’s University of Technology Thonburi, 126 Pracha Uthit Rd., Bang Mod, Thung Khru, Bangkok 10140, Thailand; 3 Assistive Technology and Medical Devices Research Center (A-MED), National Science and Technology Development Agency, Pathum Thani 12120, Thailand; 4 National Metal and Materials Technology Center (MTEC), National Science and Technology Development Agency, Pathum Thani 12120, Thailand; 5 Division of Biomaterials and Tissue Engineering, UCL Eastman Dental Institute, Royal Free Hospital, Rowland Hill Street, London NW3 2PF, UK; 6 Thammasat University Research Unit in Dental and Bone Substitute Biomaterials, Thammasat University, Pathum Thani 12120, Thailand

**Keywords:** resin-modified glass ionomer cement, flexural strength, polymerization, bioactive glass, calcium phosphates

## Abstract

This study prepared low-toxicity, elemental-releasing resin-modified glass ionomer cements (RMGICs). The effect of 2-hydroxyethyl methacrylate (HEMA, 0 or 5 wt%) and Sr/F-bioactive glass nanoparticles (Sr/F-BGNPs, 5 or 10 wt%) on chemical/mechanical properties and cytotoxicity were examined. Commercial RMGIC (Vitrebond, VB) and calcium silicate cement (Theracal LC, TC) were used as comparisons. Adding HEMA and increasing Sr/F-BGNPs concentration decreased monomer conversion and enhanced elemental release but without significant effect on cytotoxicity. Rising Sr/F-BGNPs reduced the strength of the materials. The degree of monomer conversion of VB (96%) was much higher than that of the experimental RMGICs (21–51%) and TC (28%). The highest biaxial flexural strength of experimental materials (31 MPa) was significantly lower than VB (46 MPa) (*p* < 0.01) but higher than TC (24 MPa). The RMGICs with 5 wt% HEMA showed higher cumulative fluoride release (137 ppm) than VB (88 ppm) (*p* < 0.01). Unlike VB, all experimental RMGICs showed Ca, P, and Sr release. Cell viability in the presence of extracts from experimental RMGICs (89–98%) and TC (93%) was significantly higher than for VB (4%). Experimental RMGICs showed desirable physical/mechanical properties with lower toxicity than the commercial material.

## 1. Introduction

Dental caries is a major preventable chronic disease that affects people globally [1]. It was estimated that 2 billion and 514 million people were suffering from untreated caries in permanent and in primary teeth, respectively. If left untreated, caries lesions may rapidly progress, leading to extensive cavities that require restorative treatment. The current minimally invasive restorative approach for deep caries involves selective caries removal, in which soft or firm dentin is left over the pulp to preserve the dentin-pulp complex. This may subsequently reduce the risk of pulpal complications [2]. Currently, there is no strong conclusive evidence supporting the placement of pulp protection materials, such as base or liner, over infected dentin [3,4,5]. However, some clinicians may still prefer to place ion-releasing materials, such as resin-modified glass ionomer cements (RMGICs), over the remaining caries [6].

The main attractive properties of RMGICs include command setting through free-radical polymerization in addition to acid–base reaction and the ability to release fluoride to promote tooth remineralization. Moreover, these materials contain methacrylate monomers, such as 2-hydroxyethyl methacrylate (HEMA). These promote wetting of moist dentine [7] and—through polymerization—bonding with resin composites. However, the release of residual HEMA monomers demonstrated a cytotoxic effect [8,9,10], thus limiting the placement of RMGICs directly on deep cavities with suspected pulp exposure.

Previous studies prepared RMGICs by using polyacrylic acids functionalized with methacrylate groups [11,12]. The powder phase contained pre-reacted fluoroaluminosilicate glass to help enhance the glass–polymer interaction [13]. These materials exhibited acceptable rheological properties with slightly lower shear bond strength (10 MPa) to dentine compared with the commercial material (Vitrebond) (16.5 MPa) [11]. However, the experimental materials demonstrated superior relative cell viability for dental pulp cells (82–89%) when compared to a commercial RMGIC (55%) [12]. The major limitation of the RMGICs was their setting reactions and fluoride release. Potiprapanpong et al. [12] showed that the amount of fluoride released from RMGICs containing polyacrylic acids functionalized with methacrylate groups was approximately half of that observed from the commercially available RMGIC. Adding phosphopeptide-amorphous calcium fluoride phosphate nanocomplex (CPP-ACFP) to a commercial conventional GIC enabled the release of calcium (Ca), inorganic phosphate (Pi), and fluoride ions [14]. The concern with CPP-ACFP particles is the reduction in physical/mechanical properties of materials due to their high hydrophilicity [15]. A previous study incorporated spherical Sr-bioactive glass nanoparticles into resin-based orthodontic adhesives and dental sealants [16,17]. The additive promoted Ca, Sr, and P ion release, which enhanced mineralizing actions and growth inhibition of planktonic *S. mutans* without detrimental effects on the mechanical properties of the materials [16]. The addition of Sr/F-bioactive glass nanoparticles (Sr/F-BGNPs) in the current study was expected to enhance ion release for RMGICs. A study showed that incorporating 45S5 bioactive glass into GIC enhanced mechanical properties and mineralizing actions of the materials [18]. However, the strength decreased when increasing the concentration of 45S5 bioactive glass due to the excessive debonding of fillers from the matrix phase.

This study therefore aimed to prepare low-toxicity RMGICs containing polyacrylic acids functionalized with methacrylate groups, with or without a low concentration of 2-hydroxyethyl methacrylate (0 or 5 wt% HEMA). The powder phase was added with spherical Sr/F-bioactive glass nanoparticles (5 or 10 wt% Sr/F-BGNPs), which was expected to enhance ion release for the materials. The objectives were to assess setting reaction, biaxial flexural strength/modulus, ion release, and in vitro cytotoxicity of the experimental RMGICs compared with a commercial material. The effects of increasing HEMA and Sr/F-BGNPs concentrations on the tested properties were examined. The null hypotheses of this study were as follows: (i) experimental RMGICs exhibit comparable polymerization/mechanical properties and in vitro cytotoxicity to commercial pulp protection materials, and (ii) an increase in HEMA or Sr/F-BGNPs concentrations has no significant effects on the tested properties of the experimental materials.

## 2. Results

### 2.1. Assessment of Setting Reaction

**Figure 1:**
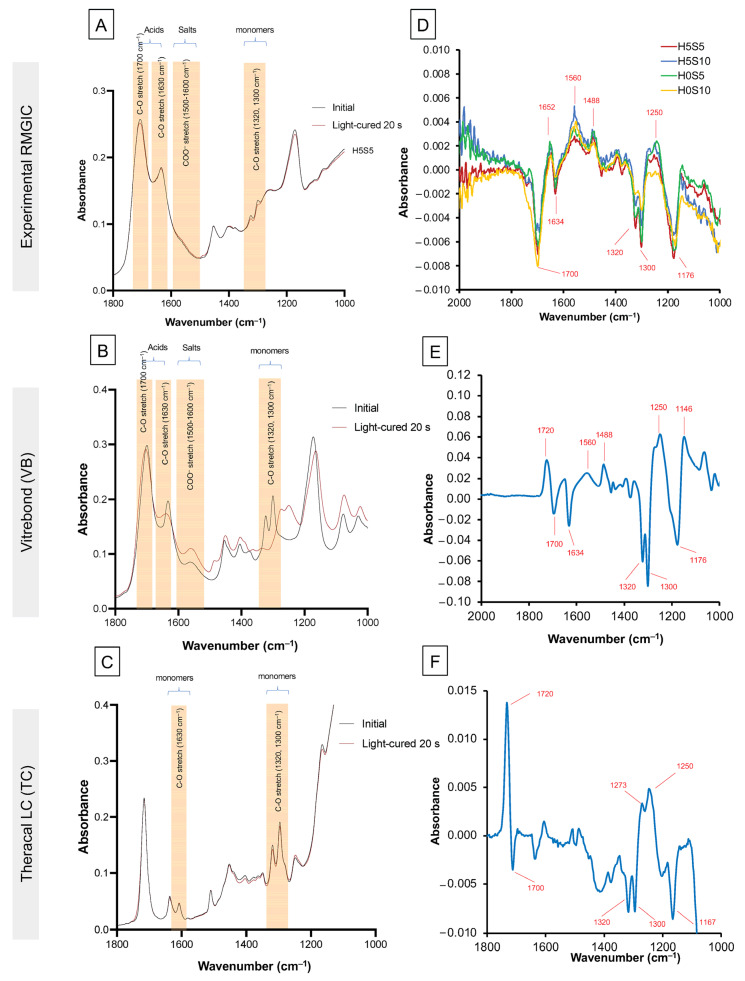
Absorbance and absorbance change after light-curing for the (**A**) experimental RMGICs, (**B**) VB, and (**C**) TC. The absorbance changes spectra for (**D**) experimental RMGICs, (**E**) VB, and (**F**) TC were obtained by subtracting initial spectra from those after light-curing for 20 s.

**Figure 2:**
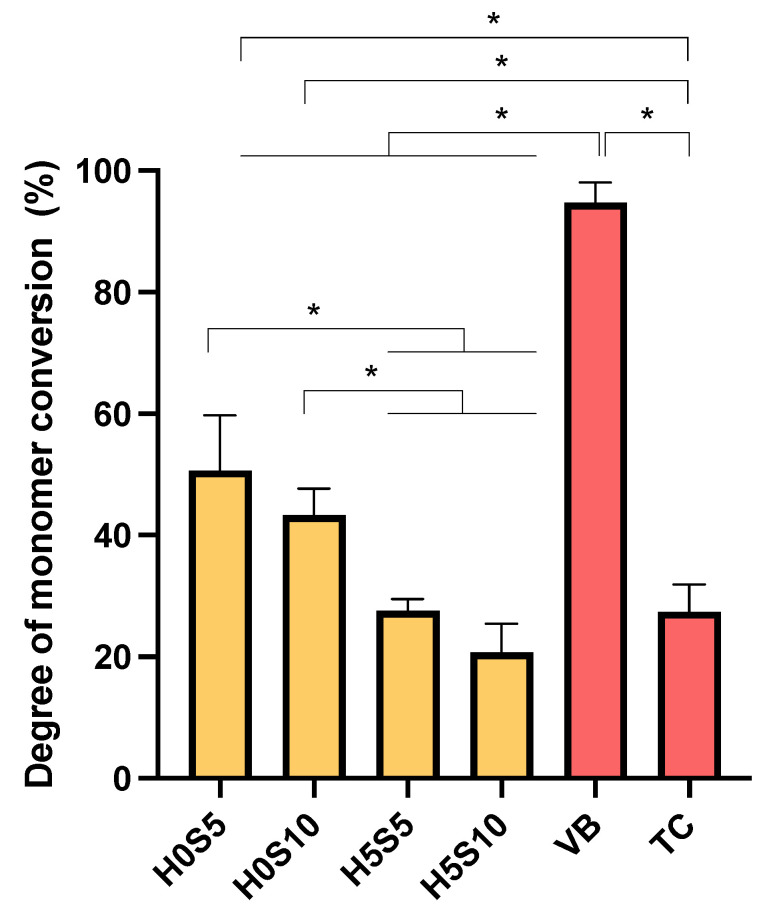
Degree of monomer conversion of all materials after light-curing for 20 s. Error bars are SD (*n* = 4). Asterisks (*) indicate *p* < 0.05.

Example FTIR (Fourier-transform infrared) spectra of the experimental RMGICs before and immediately after 20 s of light exposure are shown in Figure 1A. Those for VB and TC are provided in Figure 1B,C, respectively. The complete loss of the C-O peak at 1320 cm^−1^ for VB suggests close to 100% (96 ± 2%) conversion (Figure 2). Conversely, with the experimental RMGICs and TC, this methacrylate C-O peak is still detectable after light activation (Figure 1A,C). For TC, the level of conversion is calculated to be 28 ± 4%. The DC of VB was significantly higher than that of all experimental RMGICs (*p* < 0.01). H0S5 (51 ± 9%) showed a comparable DC to H0S10 (43 ± 4%) (*p* = 0.2084), but both formulations were significantly higher than H5S5 (28 ± 2%) and H5S10 (21 ± 5%) (*p* < 0.05). Factorial analysis indicated that the addition of HEMA showed a reduction in DC by 49 ± 6%. Additionally, the increase in Sr/F-BGNPs concentrations reduced DC by 19 ± 16%.

Difference spectra, obtained by subtracting the initial spectra from those at 20 s after light exposure, are shown in Figure 1D–F for the experimental RMGICs, VB, and TC, respectively. All materials show peaks and troughs in these difference spectra at similar wavenumbers consistent with polymerization, causing all changes. The trough at 1640 cm^–1^ is expected upon loss of the methacrylate C=C. Other changes in the 1500 cm^–1^ and 1750 cm^–1^ regions are likely due to changes in the environment and stretching of the methacrylate C=O. Changes between 1350 cm^–1^ and 1500 cm^–1^ would have likely been due to the methacrylate CH_2_ groups changing their vibrational modes significantly when the monomer polymerizes. The shift in the doublet at 1320/1300 to 1270/1250 cm^–1^ occurs due to changes in the methacrylate C-O group vibration. The level of change in difference spectra during polymerization for the experimental RMGICs was approximately 10 times smaller than those seen with VB. This was likely due to the combined lower percentage conversion and initial concentration of methacrylate groups in the materials.

### 2.2. Biaxial Flexural Strength (BFS) and Modulus (BFM)

**Figure 3:**
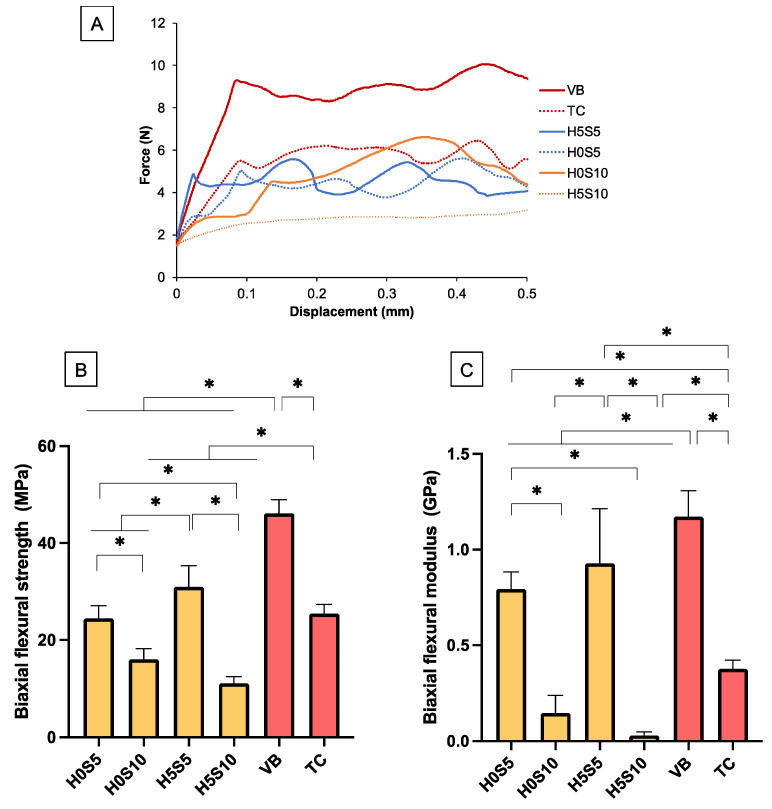
(**A**) Force versus displacement curves of representative samples from the BFS test. (**B**) Biaxial flexural strength and (**C**) biaxial flexural modulus of materials. Error bars are SD (*n* = 5). Asterisks (*) indicate *p* < 0.05.

The force–displacement diagram showed that the experimental RMGICs yielded at a lower level of applied force than VB (Figure 3A). The highest BFS was detected with VB (46 ± 3 MPa), whereas the lowest was obtained from H5S10 (11 ± 1 MPa) (Figure 3B). The BFS of VB was significantly higher than other materials (*p* < 0.05). The BFS of TC (25 ± 2 MPa) was significantly lower than that of H5S5 (31 ± 4 MPa) (*p* < 0.01). H5S5 showed significantly higher BFS than H0S5 (24 ± 3 MPa), H0S10 (16 ± 2 MPa), and H5S10 (*p <* 0.05). For BFM (Figure 3C), the highest and lowest values were also obtained from VB (1.17 ± 0.12 GPa) and H5S10 (0.03 ± 0.02 GPa). The BFM of TC (0.38 ± 0.05 GPa) was significantly lower than that of H0S5 (0.79 ± 0.09 GPa) and H5S5 (0.93 ± 0.29 GPa). H5S5 exhibited a significantly higher BFM than H0S5, H0S10 (0.15 ± 0.09 GPa), and H5S10.

**Figure 4:**
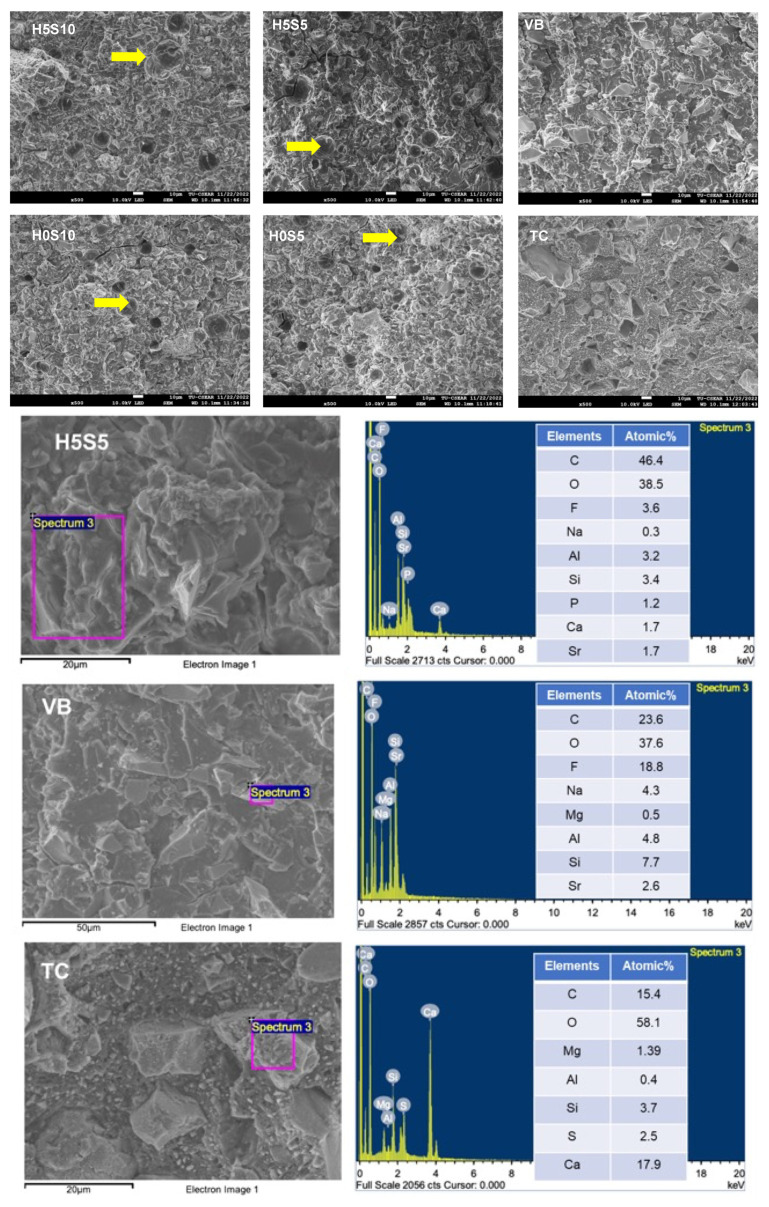
Fracture surface of the tested specimens. Multiple voids that could be due to air bubble entrapment were mainly detected with the experimental RMGICs (arrows). EDX result of tested specimens. A representative sample from experimental RMGIC (H5S5) and VB showed the fracture surface containing elements. Ca can be detected with TC.

Factorial analysis showed that the addition of HEMA and increasing the Sr/F-BGNPs level reduced BFS and BFM by 6 ± 2% and 51 ± 7%, respectively. Moreover, BFM was reduced by 52 ± 24% and 92 ± 4% upon an increase in concentrations of HEMA and Sr/F-BGNP, respectively. The fracture surface of experimental RMGICs and VB showed remaining glass fillers embedded in the matrix (Figure 4). However, multiple pores were detected with the experimental materials. SEM of TC showed fillers which could be Ca-Si particles.

### 2.3. Elemental Release

**Figure 5:**
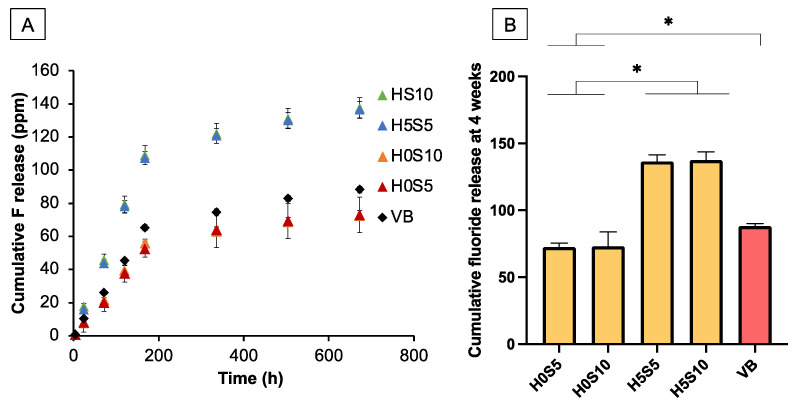
(**A**) Cumulative fluoride release from all materials upon immersion in water for up to 4 weeks. (**B**) Average cumulative fluoride release at 4 weeks. Error bars are SD (*n* = 5). Asterisks (*) indicate *p* < 0.05.

No fluoride release was detected from TC. For other materials, the cumulative release of fluoride was initially proportional to time (h) (Figure 5A). The highest cumulative fluoride release at the late time (Figure 5B) was detected with H5S10 (137.5 ± 6.1 ppm), which was comparable to that of H5S5 (136.6 ± 2.2 ppm) (*p* > 0.05). Both were significantly higher than that of VB (88.3 ± 1.9 ppm), H0S10 (72.6 ± 3.0 ppm), and H0S5 (73.0 ± 9.4 ppm) (*p* < 0.05). The fluoride release of H0S10 was not significantly different from that of H0S5 (*p* = 0.991), but these were lower than that of VB (*p* < 0.05). Factorial analysis showed that the use of HEMA enhanced the average cumulative fluoride release by 89 ± 11%, but the effect of raising the Sr/F-BGNPs level was minimal.

The highest release of Al was detected with H5S10 (Table 1). That obtained from H5S10 was significantly higher than that of H5S10 (*p* = 0.0019) and H0S5 (*p* = 0.0006). The highest Ca release was from TC. This was significantly higher than for all other materials (*p* < 0.05). P release was only observed with the experimental RMGICs. H5S10 exhibited significantly higher P release than H5S5 (*p* = 0.0008) and H0S5 (*p* = 0.001). The level of P release from H0S10 was comparable to that of H5S10 (*p* = 0.0904). For Sr, H5S10 exhibited significantly higher Sr release compared with H5S5 (*p* = 0.0127) and H0S5 (*p* = 0.0096).

Factorial analysis showed that the addition of HEMA enhanced the release of Al, Ca, P, and Sr ions by 38 ± 23%, 53 ± 21%, 42 ± 12%, and 36 ± 18%, respectively. Additionally, the increase in Sr/F-BGNPs enhanced the release of Al, Ca, P, and Sr by 167 ± 36%, 152 ± 36%, 124 ± 15%, and 137 ± 35%, respectively.

### 2.4. Assessment of Cytotoxicity

**Figure 6:**
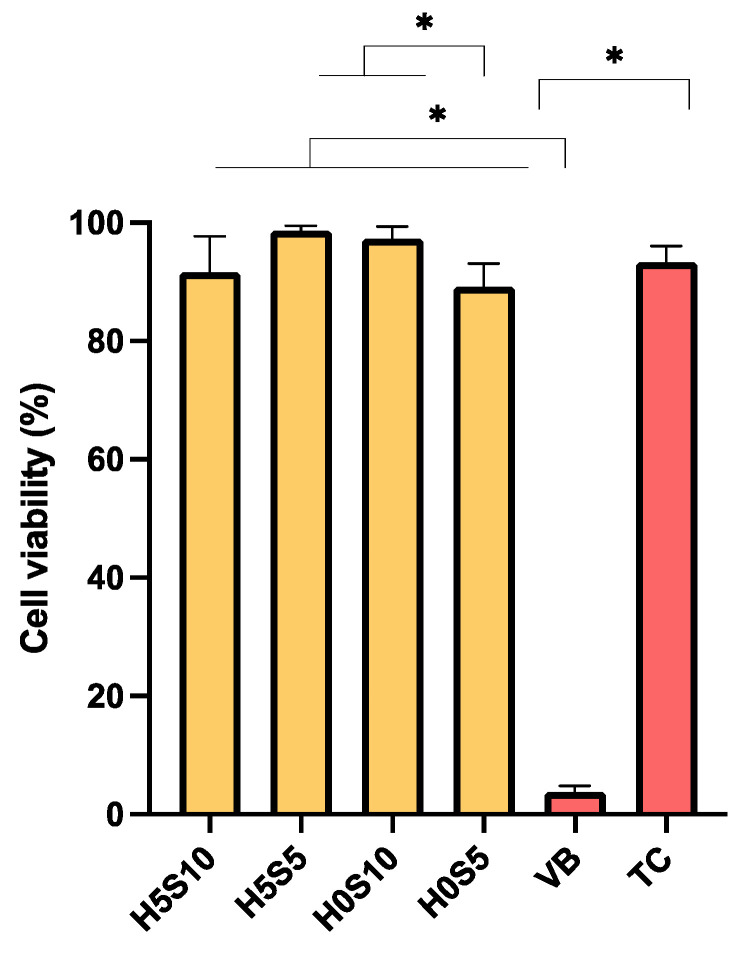
Cell viability in the presence of material extracts. Percentage of cell viability of mouse fibroblasts after exposure to extracts from each material. Error bars are SD (*n* = 3). Asterisks (*) indicate *p* < 0.05.

Cell viability in the presence of extracts was highest for H5S5 (99 ± 1%) and lowest with VB (4 ± 1%) (Figure 6). The cell viability of TC (93 ± 3%) was comparable to that of H5S10 (92 ± 6%), H5S5, H0S10 (97 ± 2%), and H0S5 (89 ± 4%) (*p* > 0.05). Factorial analysis showed that both the addition of 5 wt% HEMA and increasing Sr/F BGNPs showed minimal effects on cell viability.

## 3. Discussion

The aim of this research was to prepare RMGICs that exhibited low toxicity but provided a high level of fluoride release. The first hypothesis was partially rejected as the experimental RMGICs exhibited setting mechanisms comparable to the commercial RMGIC, but the experimental materials showed lower strength than the commercial material. The addition of HEMA and Sr/F-BGNPs also significantly influenced biaxial flexural strength and elemental release. Hence, the second null hypothesis was also rejected. It should be noted that the current study is an in vitro study. Therefore, its clinical implications should be carefully interpreted.

Minimal changes of setting via acid–base reaction and free radical polymerization were detected with experimental RMGICs. The use of HEMA at low concentration in combination with methacrylate functionalization produced a shallow peak at 1320 cm^–1^. The results, however, confirmed that the experimental RMGICs could be set through light-activated polymerization. The minimal light-activated polymerization could be advantageous by allowing acid–base neutralization and water sorption for ion-releasing actions. However, limited light-activated polymerization may inevitably result in low initial strength of the materials [19]. It was expected that the continuation of an acid–base reaction may lead to the maturation of the RMGICs and increase their strength over time [20]. The experimental RMGICs showed lower DC than the commercial RMGIC. This finding was in agreement with that of the previous study [12]. DCs observed with formulations in the current study was slightly greater than with the original formulations. It was speculated that the mixture of fluoroaluminosilicate glass and Sr/F-BGNPs may affect optical properties and light penetration [21]. This should be examined in future work.

The reduction of the 1320 cm^–1^ peak was also observed with TC. Previous studies suggested that the limited polymerization of TC may be attributable to the high opacity of Ca-Si cements that reduces light transmission into the deep parts of TC [12,22]. Another cause of low TC could be due to the use of high glass transition temperature monomers such as Bis-GMA [23]. This may affect the use of TC if applied in a thick layer. The limitation of the current study was that the measurement only focused on the initial time (20 s after light curing). It was expected that the materials may undergo maturation and setting processes over time, which shall be examined in future work.

The experimental RMGICs exhibited inferior mechanical properties compared with other materials. However, the flexural strength of experimental materials in the current study was still higher than the 10 MPa required by the ISO 9917-2:2017: Dentistry—Water-based cements-Part 2: Resin-modified cements [24]. The strength and modulus of the experimental RMGICs in the current study were much lower than those in previous studies (BFS = 30–40 MPa, modulus = 1–2 GPa), which could be due to the addition of Sr/F-BGNPs. The low rigidity of RMGICs caused by the addition of 10 wt% Sr/F-BGNPs may additionally reduce the precision of the strength measurement in the current study.

Sr/F-BGNPs lack the ability to enable acid–base neutralization to aid the forming of silica hydrogel and crosslinking with polymeric acids. The lack of silanization of Sr/F-BGNPs may also limit the bonding potential with the methacrylate groups in HEMA or the modified polyacrylic acids. The silanization may reduce the ion-releasing ability of the bioactive glass. However, a recent study demonstrated that the use of silanized bioactive glass in dental adhesive showed a significant effect on mechanical properties and remineralizing effects on dentin. Future work may employ a silanization technique for treating a surface of Sr/F-BGNPs [25].

The formulations that contained 5 wt% Sr/F-BGNPs showed satisfactory BFS and BFM. Raising the HEMA level enhanced the BFS of the materials when the level of Sr/F-BGNPs was low (5 wt%). A possible explanation could be that the addition of HEMA reduces the viscosity of the materials. This may promote ionization or movement of acids to react with fluoroaluminosilicate glass, enhancing crosslinking between glass and acids. The long-term mechanical properties should be examined in future work.

The addition of Sr/F-bioactive glass nanoparticles (Sr/F-BGNPs) was expected to increase the release of fluoride. The modified formulations of RMGICs in the current study successfully enabled a higher level of fluoride release compared with commercial material and the original formulations in the previous study [12]. The high surface area of Sr/F-BGNPs may enable rapid reaction with absorbed water leading to the dissolution of the glass network and ion release from the specimens. Another possible reason may be that the space or interfacial gaps (due to the lack of chemical adhesion between non-silanized RMGICs) promoted the diffusion of fluoride [26]. The low modulus of elasticity observed with experimental RMGICs may additionally promote the release of ions. Moreover, the fluoride release profile for up to 1 week can be explained using the following equation [27]:

(1) $$\left[F_{c}\right]=\frac{{\left[F\right]}_{1}t}{t+t_{\frac{1}{2}}}+\beta \sqrt{t}$$

where the first term represents the release of fluoride from the early washout process, which could take approximately two weeks [28]. This was in accordance with the current study, which shows the release of fluoride that is directly proportional to immersion time. The current study showed that the addition of HEMA significantly promoted ion release. This was in agreement with the previous study [12]. However, the increase in Sr/F-BGNPs concentration resulted in no significant effect on the release of fluoride. It was hypothesized that the degree of glass dissolution may be primarily controlled by the flexibility of the matrix rather than the concentration of ion-releasing fillers. Additionally, the amount of fluoride released may be mainly governed by the glass ionomer phase in the material.

All experimental RMGICs demonstrated the ability to release other elements apart from fluoride, including Ca, Sr, Al, and P. The release was strongly enhanced by the addition of HEMA. This may be due to water sorption helping to plasticize the polymer network leading to the enhancement of ion diffusion and release. Future work should assess the remineralizing effects of experimental RMGICs on demineralized dentin.

According to BS EN ISO 10993-5:2009, the lowest relative cell viability considered as having cytocompatibility was 70% [29]. This may indicate that the experimental RMGICs and TC would pass this requirement and be considered to exhibit low toxicity compared to VB. This was also in agreement with the previous study [12]. Furthermore, the low cell viability in the presence of VB extracts was in accordance with a previous study [30]. This could be attributed to the release of HEMA and the acidity of the extract [31]. The similar cell viability of the experimental RMGICs to TC could be due to the use of HEMA at low concentration (5 wt%). This is supported by a previous study that found a significant reduction in dental pulp cell viability when exposed to an extract of dental adhesive containing 10 or 20 wt% HEMA [32]. Furthermore, it was expected that the ionic products from bioactive glass degradation may additionally promote cell viability, enhance mitochondrial activity, and induce cell proliferation [33]. The release of phosphate from bioactive glass nanoparticles may help neutralize the low pH, which is usually observed with GICs that release high levels of fluoride.

The limitation of the current study is the lack of analysis of the eluate. The identification of compounds in the eluate by HPLC and pH measurement may be needed in future work. The cytocompatibility of the experimental RMGICs was expected to expand their application in deep cavities with suspected pulp exposure (indirect pulp capping).

Within the scope of this study, the RMGIC formulation containing 5 wt% HEMA and 5 wt% Sr/F-BGNPs (H5S5) exhibited adequate mechanical properties, desirable elemental release, and appropriate cytotoxicity effects. Further studies may assess the performance of H5S5 on dentin remineralization, cell mineralization, antibacterial actions, and bonding performance with both dentin and resin composite.

## 4. Materials and Methods

### 4.1. Preparation of Liquid Phase

**Figure 7:**
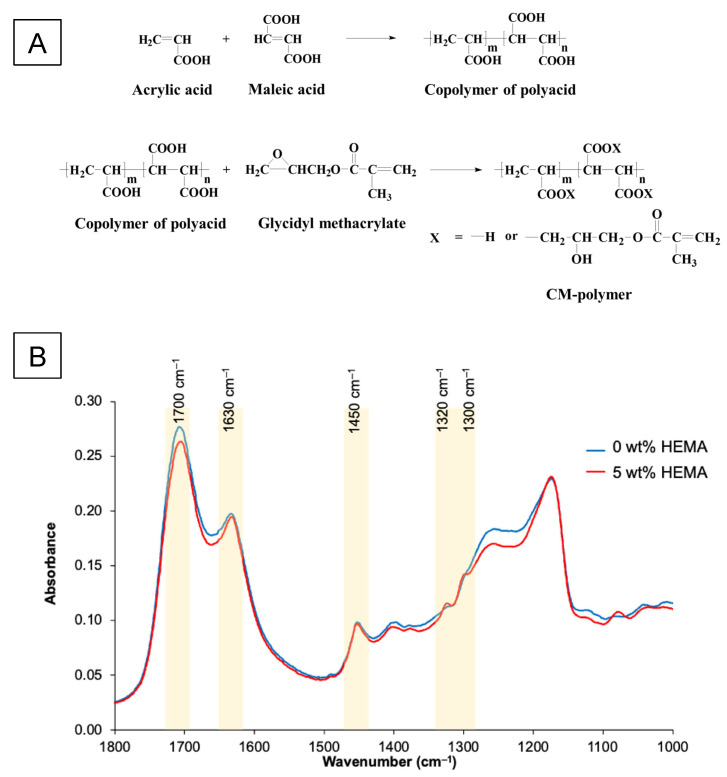
(**A**) The synthesis of polyacrylic acids functionalized with methacrylate groups (CM polymer). (**B**) FTIR spectra of liquid phase (CM copolymer in water) containing 0 or 5 wt% HEMA.

The main component of the liquid phase was methacrylate-functionalized acrylic acid/maleic acid copolymer (CM copolymer) (Figure 7A). The copolymer was synthesized in an aqueous solution using a 4:1 feed molar ratio of acrylic acid (AA, Acros Organics, Fair Lawn, NJ, USA) to maleic acid (MA, Sigma-Aldrich, St. Louis, MO, USA) or 20% *v*/*v* of AA to 10% *w*/*v* of MA. Potassium persulphate (3% *w*/*v*, Honeywell Fluka, Charlotte, NC, USA) and isopropanol (13% *v*/*v*, RCI Labscan, Bangkok, Thailand) were utilized as an initiator and a chain-transfer agent, respectively. The reaction was performed under a nitrogen atmosphere at 80 °C for 4 h. The obtained copolymer was concentrated using a rotary evaporator (BUCHI Rotavapor R-114, BUCHI, Flawil, Switzerland) and purified using a dialysis membrane with a molecular weight cut-off of 3.5 kDa in deionized water for 48 h. The copolymer was then freeze-dried using a lyophilizer (Supermodulyo-230, Thermo Fisher Scientific, Waltham, MA, USA). The AA/MA molar ratio of the resultant copolymer was 3.2:1, as determined by proton nuclear magnetic resonance spectroscopy (^1^H NMR, Bruker DPX-300 spectrometer, Bruker BioSpin, Rheinstetten, Germany). The weight average molar mass of the copolymer determined by Gel Permeation Chromatography (GPC, Water 600E, Waters, Milford, MA, USA) was approximately 37,500 Dalton with a polydispersity index of 1.68.

The AA/MA copolymer (14% *w*/*v*) was further reacted with 8.5% *v*/*v* glycidyl methacrylate (GMA, Sigma-Aldrich, St. Louis, MO, USA) using 0.1% *v*/*v* pyridine (RCI Labscan Limited, Bangkok, Thailand) as a catalyst and 0.07% *w*/*v* butylated hydroxytoluene (Honeywell Fluka, Charlotte, NC, USA) as an inhibitor. The reaction was carried out in tetrahydrofuran at 60 °C for 8 h under nitrogen gas and then kept at room temperature overnight. The methacrylate-functionalized copolymer was precipitated in diethyl ether and dried in a vacuum oven at room temperature. The degree of methacrylation, which was analyzed using 1H NMR (Ascend TM 600/Avance III HD, Bruker, Billerica, MA, USA), was 0.1 mole per mole of an acid group in the copolymer.

Functionalized polyacid (55 or 50 wt%) was mixed with 45 wt% water and either 0 or 5 wt% 2-hydroxyethyl methacrylate (HEMA, Sigma-Aldrich, St. Louis, MO, USA). To both, 2 pph of tartaric acid (Sigma-Aldrich, St. Louis, MO, USA), 0.7 pph of camphorquinone (CQ, Sigma-Aldrich, St. Louis, MO, USA), and 1.4 pph of N,N’-dimethylaminoethyl methacrylate (DMAEMA, Sigma-Aldrich, St. Louis, MO, USA) were then added. FTIR spectra (Nicolet iS5, Thermo Fisher Scientific, Waltham, MA, USA) of these liquids had weak methacrylate peaks due to C-O stretching (1300 and 1320 cm^−1^, respectively) (Figure 7B).

### 4.2. Preparation of Powder Phase

#### 4.2.1. Preparation of Pre-Reacted Fluoroalumino Silicate Glass

**Figure 8:**
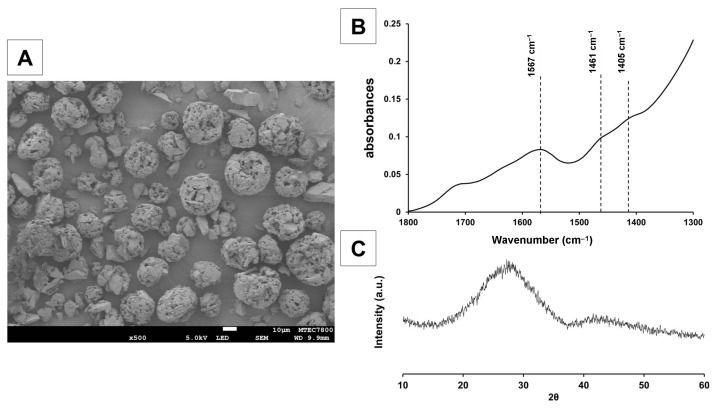
Characteristics of pre-reacted fluoroaluminosilicate glass: (**A**) scanning electron microscopy (SEM) image of the fluoroaluminosilicate glass, (**B**) FTIR spectra, and (**C**) XRD pattern of the pre-reacted fluoroaluminosilicate glass.

Fluoroaluminosilicate glass (SiO_2_-Al_2_O_3_-CaF_2_-ZrO_2_) was prepared by mixing SiO_2_ (Ajax Finechem, Thermo Fisher Scientific, Waltham, MA, USA), Al_2_O_3_ (Fluka Analytical, Honeywell Fluka, Charlotte, NC, USA), P_2_O_5_ (ACROS ORGANICS, Thermo Fisher Scientific, Waltham, MA, USA), CaF_2_ (Merck, Darmstadt, Germany), ZrO_2_ (Sigma-Aldrich, St. Louis, MO, USA) and SrCO_3_ (Sigma-Aldrich, St. Louis, MO, USA) (Table 2). These powders were melted in a Pt-10% Rh crucible at 1450 °C for 2 h. The melted glass was rapidly quenched in water to produce glass frits. The obtained frits were milled using a planetary micromill (Fritsch Pulverisette 7, FRITSCH, Idar-Oberstein, Germany), followed by ball-milling to achieve a particle size (D_0_._5_) around 5 μm, which was confirmed by A laser diffraction technique (Mastersizer 2000, Malvern Instruments, Malvern, UK). Additionally, X-ray diffraction (XRD, PANalytical, Malvern Panalytical, Malvern, UK) was used to confirm the amorphous nature of the glass (Figure 8C). This was operated from 20–60° 2θ at a scan speed of 2° 2θ/min and a step size of 0.02° 2θ with CuK_α_ radiation (K_α_ = 1.5406 nm) at 30 mA and 50 kV.

The pre-reacted fluoroalumino silicate glass was prepared to enhance the mechanical properties of the materials [13]. The pre-reacted glass was prepared by mixing the above glass powder with deionized water at 3:7 *w*/*w* with 2 wt% of 55 wt% aqueous CM to obtain a slurry mixture. The slurry was then spray-dried to produce pre-reacted glass fillers. The FTIR spectra confirmed the presence of peaks attributed to polyacrylate salts (symmetric COO-stretch, 1470–1400 cm^−1^; asymmetric COO-stretch, 1600–1500 cm^−1^) that resulted from pre-reaction of polyacid and basic glass fillers (Figure 8A,B).

#### 4.2.2. Preparation of Sr/F Bioactive Glass Nanoparticles (Sr/F-BGNPs)

Spherical bioactive glass nanoparticles with a diameter of 160 ± 20 nm were synthesized through a sol–gel process and post-functionalization. The silica nanoparticles (SiO_2_-NPs) were first prepared prior to the incorporation of calcium (Ca), strontium (Sr), sodium (Na), and fluoride (F) through a heat treatment process. Firstly, 329.2 mL of ethanol (Merck, Darmstadt, Germany), 41.1 mL of deionized water, and 4.8 mL of ammonium hydroxide (Merck, Darmstadt, Germany) were mixed in a 1 L Erlenmeyer flask. This was stirred at a rate of 600 rpm using a magnetic stirrer for 15 min. Then, 25.0 mL of tetra orthosilicate (TEOS, Sigma-Aldrich, St. Louis, MO, USA) was gently added into the prepared solution and stirred for 16 to 18 h at room temperature to complete the hydrolysis and poly-condensation reactions. SiO_2_-NPs were collected using centrifugation at a speed of 5000 rpm at 25 °C for 30 min and re-suspended in deionized water. A total of 8.6 g of Ca(NO_3_)_2_·6H_2_O (Sigma-Aldrich, St. Louis, MO, USA), 23.1 g of Sr(NO_3_)_2_ (Merck, Darmstadt, Germany), and 0.6 g of NaF (Merck, Darmstadt, Germany) were doped into the SiO_2_-NPs using a nominal molar ratio of SiO_2_:CaO:SrO:NaF of 1.0:0.33:0.98:0.5 via the post-functionalization process. Particles were then collected and dried at 60 °C in the oven overnight to remove excess water before calcination at 680 °C. The condition of the furnace was set at a heating rate of 3 °C/min from room temperature to 680 °C and then heated at 680 °C for 3 h to remove nitrate precursors and to obtain Sr/F-BGNPs. The Sr/F-BGNPs were washed with ethanol twice prior to use.

**Figure 9:**
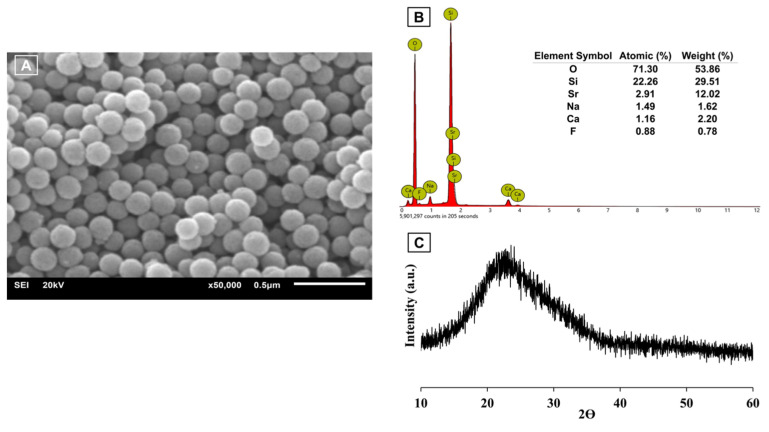
Characteristics of Sr/F bioactive glass nanoparticles: (**A**) SEM image of the particles, (**B**) EDX, and (**C**) XRD pattern of the glass.

SEM (JEOL, JSM-6610 LV, Tokyo, Japan) confirmed the Sr/F-BGNPs particles were spherical and monodispersed (Figure 9A). The particle size range, analyzed using the image analysis program (ImageJ, U. S. National Institutes of Health, Bethesda, MD, USA) (*n* = 50), was 140 to 180 nm. Ca, Sr, Na, and F were detected in EDX analysis (Energy Dispersive X-ray, OXFORD, INCAx-act, Oxford, UK) (Figure 9B). The XRD spectrum (Bruker AXS/D8Discover, Bruker BioSpin, Switzerland) exhibited a broad halo at 2θ between 20–30°, indicative of an amorphous phase (Figure 9C). Taken together, Ca, Sr, Na, and F were successfully incorporated into the silica network whilst maintaining the monodispersity of the spherical nanoparticles, and their amorphous nature.

### 4.3. Preparation of Experimental RMGICs

Four experimental formulations of RMGICs were prepared (Table 3). The powder and liquid were mixed using a powder-to-liquid ratio (PLR, mass ratio) of 1.5:1. The powder and liquid were weighed using a four-figure analytical scale and mixed by hand using a spatula for 10 s. The commercial pulp protection materials, including RMGIC (Vitrebond; 3M, 3M ESPE, St. Paul, MN, USA) and Ca-Si cement (Theracal LC; Bisco, Schaumburg, IL, USA) were used as comparisons (Table 4).

### 4.4. Assessment of Setting Reaction

ATR-FTIR was used to determine the setting reaction of materials upon light activation (*n* = 4). Samples were prepared and placed on the ATR diamond. The sample was surrounded by a metal circlip (1 mm thick) and covered by a clear acetate sheet. These were then light-cured using an LED light-curing unit for 20 s from the top. The tip was positioned at approximately 4 mm above the specimen’s surface to mimic the placement of the material in a deep caries cavity. FTIR spectra of the bottom surface of the specimens were recorded before and after light-curing. The DC was calculated using the following equation [34]:

(2) $$DC=\frac{100(∆A_{0}-∆A_{t})}{∆A_{0}}$$

where $∆A_{0}$ and $∆A_{t}$ are the height of the peak at 1320 cm^–1^ (C-O of methacrylate group) [35] above the background level of 1335 cm^–1^ before curing and at time t after initiating curing, respectively. For experimental RMGICs, the height of the peak was determined above the baseline between 1314 cm^–1^ and 1335 cm^–1^. The FTIR peaks of PAA (carboxylic acids) were 1720 cm^–1^ (C=O stretch), 1452 cm^–1^ (C-H scissor), 1370 cm^–1^ (C-H bend), 1249 cm^–1^ (C-O stretch) [36]. Additionally, the FTIR peaks representing monomers were 1700 cm^–1^, 1635 cm^–1^ (C=C stretch), 1452 cm^–1^, 1370 cm^–1^, 1320 cm^–1^ (C-O stretch), 1300 cm^–1^ (C-O stretch). The FTIR peaks for glass fillers and polyacrylte salts were 1000 cm^–1^ (Si-O stretch) and 1460 cm^–1^ to 1600 cm^–1^, respectively.

### 4.5. Assessment of Biaxial Flexural Strength (BFS) and Modulus of Elasticity (BFM)

Biaxial flexural strength and modulus of elasticity (*n* = 5) were determined using a ball-on-ring testing jig (the diameter of the ball and internal diameter of the supporting ring are 4 mm and 8 mm, respectively). Materials were mixed and placed in a metal ring (10 mm internal diameter and 1 mm in thickness). The specimens were then cured using an LED light curing unit for 40 s on both the top and bottom surfaces. The specimens were left at room temperature for 24 h before immersion in 10 mL of deionized water. They were incubated at 37 °C in an incubator for an additional 24 h prior to the test. The thicknesses were measured using a digital vernier caliper (YOUFOUND, FISCO, Yokohama, Japan). The disc specimen was placed on a testing jig under the mechanical testing frame (AGSX, Shimadzu, Kyoto, Japan), and a load of 500 N was applied on the specimen with a crosshead speed of 1 mm/min. Then, BFS and BFM were calculated using the following equations:

(3) $$BFS=\frac{F}{d^{2}}\{\left(1+v)\left[0.485\ln\left(\frac{r}{d}\right)+0.52\right]+0.48\right\}$$

(4) $$BFM=\left(\frac{∆H}{{∆W}_{c}}\right)\times \left(\frac{\beta_{c}d^{2}}{q^{3}}\right)$$

where F refers to the maximum load (N), d is the specimen’s thickness (m), r is the radius of circular support (m), v is Poisson’s ratio (0.3) [37,38], $\frac{∆H}{{∆W}_{c}}$ is the rate of change of load with regard to central deflection (N/m), β_c_ is the center deflection junction (0.5024) [39], and q is the ratio of support radius to the radius of the disc. The method for calculating β_c_ was provided in the previous study [39].

### 4.6. Assessment of Elemental Release

The cumulative release of fluoride in deionized water of all materials was determined using a fluoride-specific electrode (Orion Versastar Pro, Thermoscientific, Waltham, MA, USA) (*n* = 5). Disc specimens, prepared as above, were immersed in 10 mL of deionized water at 37 °C for up to 4 weeks. At each time point (4, 24, 72, 120, 168, 336, 504, 672 h), the specimens were removed and placed in fresh storage solution. A calibration curve was obtained using standard fluoride solution (1, 10, 100, 1000 ppm). The collected storage solution was mixed with TISAB II (Orion ionplus, Thermoscientific, Waltham, MA, USA) using 1:1 volume ratio. The amount of fluoride was then measured using the specific fluoride electrode. In addition, the amount of Al, Ca, P, and Sr at 4 weeks was analyzed using Inductively Coupled Plasma Optical Emission Spectrometry (ICP-OES, Optima 8300, PerkinElmer, Waltham, MA, USA) (*n* = 3). Wavelengths used for the detection of Al, Ca, P, and Sr were 396.153 nm, 317.933 nm, 214.914 nm, and 232.235 nm, respectively. The working range for the detection of all elements was 0.1–50 ppm. The data were presented as cumulative elemental release in deionized water.

### 4.7. Assessment of Cytotoxicity

An MTT assay was used to assess the cytotoxicity of extracts obtained from disc specimens (0.5 mm thick and 6 mm in diameter) placed in DMEM [12]. Briefly, the specimens were immersed in 200 μL of Dulbecco’s modified Eagle medium (DMEM) (Gibco, Thermo Fisher Scientific, Waltham, MA, USA) with 10% FBS (Gibco, Thermo Fisher Scientific, Waltham, MA, USA), 1% penicillin/streptomycin (Gibco, Thermo Fisher Scientific, Waltham, MA, USA) and 1% L-glutamine (Gibco, Thermo Fisher Scientific, Waltham, MA, USA) for 5 h at room temperature. Subsequently, 50 μL of the medium was added to an equal volume of fresh media (two-fold dilution) in 96-well plates.

Mouse fibroblast L292 cells were seeded at a density of 8 × 10^3^ cells/well in the prepared plates. The cells were cultured for 3 days at 37 °C and 5% CO_2_ in a humidified atmosphere. Then, the cells were incubated with 0.5 mg/mL of 3-(4,5 dimethylthiazol-2-yl)-2,5-di-phenyltetrazolium bromide (MTT) solution (invitrogen, Thermo Fisher Scientific, Waltham, MA, USA) at 37 °C for 30 min. The control was plain culture medium. The reaction was stopped with 100 μL of dimethylsulfoxide (Sigma-Aldrich, St. Louis, MO, USA) addition.

The final product’s color, determined from absorbance between 570 and 650 nm (OD, optical density), was examined using a microplate reader spectrophotometer (Varioskan^TM^ LUX Multimode, Thermo Fisher Scientific, Waltham, MA, USA). The results were reported as the relative cell viability (%) [29] by comparing with the control using the Equation (5). The assay was performed in triplicate.

(5) $$Relative\;cell\;viability=\frac{OD\;of\;the\;test\;group}{OD\;of\;the\;control}\times 100$$

### 4.8. Statistical Analysis

All reported values in this study are mean (SD). The sample size for each test was determined using G*Power version 3.1.9.6 (Heinrich-Heine-Universität Düsseldorf, Dusseldorf, Denmark). The results indicated that the number of samples employed in each test exhibited a power > 0.95 at alpha level of 0.05 for a one-way ANOVA. Data were statistically analyzed using Prism 9 for macOS (GraphPad Software version 9.5.1 for macOS, Boston, MA, USA, www.graphpad.com). The normality was assessed using the Shapiro–Wilk test. Subsequently, a one-way ANOVA was conducted, followed by a Tukey post-hoc multiple comparison test. The significance value was set at *p* = 0.05.

Additionally, factorial analysis was performed to determine the effect of HEMA and Sr/F-BGNPs levels on BFS/BFM, elemental release, and MTT test. The equation used, based on a 2 variable and 2 level factorial design, is provided in the following equation:

(6) $$lnP=\langle lnP\rangle \pm a_{1}\pm a_{2}\pm a_{1,2}$$

where $a_{1}$ and $a_{2}$ represent the effects of HEMA and Sr-BGNPs levels on the tested property (P) of the materials, respectively, and $a_{1,2}$ represents the interaction effect. The brackets indicate an average value of lnP. The percentage effect (Q) of each factor is calculated using the following equation:

(7) $$Q\left(\%\right)=100\left(1-\frac{G_{H}}{G_{0}}\right)=10(1-\exp\left(2a_{i}\right))$$

where $G_{H}$ and $G_{0}$ represent the geometric average properties (BFS, BFM, elemental release, cell viability) for the samples with high levels of HEMA and Sr-BGNPs and the samples with lower concentrations, respectively. The effect of each factor was considered significant if the value of $a_{i}$ was higher than 95% confidence interval.

## 5. Conclusions

The experimental resin-modified glass ionomer cements containing bioactive glass nanoparticles and methacrylate functionalized polyacids exhibited low in vitro toxicity and a high level of fluoride release. The use of 5 wt% HEMA with low levels of Sr/F-BGNPs enabled desirable elemental release and minimal cytotoxicity without detrimentally affecting physical/mechanical properties. Although the strength of experimental RMGICs was lower than the commercial RMGIC, the values of most formulations remain within the acceptable range.

## Figures and Tables

**Table 1:** Amount of cumulative Al, Ca, P, and Sr release (ppm) in water after 4 weeks. Data are mean (SD, *n* = 3). The same letters indicate *p* < 0.05 among different materials. NA represents a value that was lower than the detection limit of the instrument.

Materials/Element	Al	Ca	P	Sr
H0S5	0.14 (0.01) ^b,c^	0.24 (0.07) ^a^	1.08 (0.04) ^a,c^	0.41 (0.12) ^a^
H0S10	0.37 (0.08) ^c^	0.51 (0.14) ^b^	2.72 (0.23) ^c,d^	0.79 (0.24)
H5S5	0.19 (0.04) ^a^	0.31 (0.09) ^c^	1.74 (0.34) ^b,d^	0.45 (0.11) ^b^
H5S10	0.52 (0.16) ^a,b^	0.91 (0.30) ^d^	3.44 (0.48) ^a,b^	1.29 (0.39) ^a,b^
VB	0.34 (0.02)	NA	NA	NA
TC	0.33 (0.05)	25.4 (3.3) ^a,b,c,d^	NA	8.13 (0.13)

**Table 2:** Amount of cumulative Al, Ca, P, and Sr ions released (ppm) in water after 4 weeks. Data are mean (SD, *n* = 3). The same letters indicate *p* < 0.05 among different materials.

Oxides	Pre-Melted Glass Compositions (wt%)
SiO_2_	22.24
Al_2_O_3_	20.59
P_2_O_5_	12.77
SrO	22.50
CaF_2_	14.16
ZrO_2_	6.93
Total	99.19
Al_2_O_3_:SiO_2_	0.93

**Table 3:** Composition of experimental RMGICs (pph represents part per hundred).

Formulations	Liquid Phase	Powder Phase
H0S5	CM polymer (55 wt%), water (45 wt%), tartaric acid (2 pph), CQ (0.7 pph), DMAEMA (1.4 pph)	95 wt% F-Al-Si glass, 5 wt% Sr/F-BGNPs
H0S10		90 wt% F-Al-Si glass, 10 wt% Sr/F-BGNPs
H5S5	CM polymer (50 wt%), HEMA (5 wt%), water (45 wt%), tartaric acid (2 pph), CQ (0.7 pph), DMAEMA (1.4 pph)	95 wt% F-Al-Si glass, 5 wt% Sr/F-BGNPs
H5S10		90 wt% F-Al-Si glass, 10 wt% Sr/F-BGNPs

**Table 4:** Composition of commercial materials.

Formulations	Composition	Lot No.	Suppliers
Vitrebond (VB)	Liquid phase: copolymer of polyacids (35–45 wt%), HEMA (20–30 wt%), water (30–40 wt%) Powder phase: glass powder (>95 wt%), diphenyliodonium chloride (<2 wt%)	NE75067	3M ESPE, St. Paul, MN, USA
TheraCal LC (TC)	30–50 wt% Portland cement, Sr glass, fumed silica, barium sulphate, barium zirconate, bisphenol A-glycidyl methacrylate (Bis-GMA)	2200003823	Bisco Inc., Schaumburg, IL, USA

## Data Availability

The data presented in this study are available upon request from the corresponding author.
